# Pulmonary Intravascular B-Cell Lymphoma with Angiotropism/Angioinvasion Mimicking Interstitial Lung Disease: A Clinical Dilemma and Potential Diagnostic Challenge

**DOI:** 10.1155/2018/3821392

**Published:** 2018-10-08

**Authors:** Florentina Matea, Salem Alowami, Michael Bonert, Monalisa Sur, Yaron Shargall, Asghar H. Naqvi

**Affiliations:** ^1^Department of Pathology and Molecular Medicine, McMaster University, Hamilton, Ontario, Canada; ^2^Division of Thoracic Surgery, Department of Surgery, McMaster University, Hamilton, Ontario, Canada

## Abstract

Intravascular large B cell lymphoma (IVLBCL) is a rare type of extranodal diffuse large B-cell lymphoma. Patients typically present with nonspecific findings, particularly bizarre neurologic symptoms, fever, and skin lesions. IVLBCL with primary lung presentation is very rare and difficult to diagnose. The authors report a case of a 75-year-old male who presented with neurological symptoms and showed diffuse pulmonary ground glass opacities on computed tomography scan (CT scan). Surgical lung biopsy was performed. Light microscopic examination of the specimen showed diffuse alveolar septal widening caused by neoplastic lymphocytes, which were positive for CD20. These atypical lymphoid cells also demonstrated angiotropism/angioinvasion of the medium sized pulmonary vessels. The patient was diagnosed with IVLBCL and underwent chemotherapy. The patient is still alive 12 months after diagnosis.

## 1. Case Presentation

A 75-year-old male presented to the hospital with a 2-month history of decreased leg strength bilaterally, weakness and urinary retention. He had an extensive workup, including MRI of the head and spine and lumbar puncture. MRI showed multiple, nonspecific, small, scattered supratentorial white matter T2 hyperintensities worrisome for demyelination within the brain and also in the spine.

**Figure 1:**
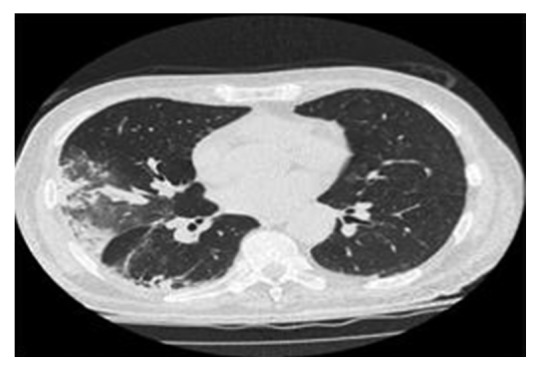
Chest CT scan showing bilateral ground glass opacities and nodular densities.

He was clinically diagnosed with transverse myelitis and started on methylprednisolone. His symptoms improved following this treatment, and he was doing well. While on the rehabilitation ward, he began to have medical issues requiring further assessment. These included a macular rash over his lower extremities and back, anaemia, cognitive decline, lymphadenopathy, and lung infiltrates which were found on chest X-ray. He underwent bronchoscopy with bronchoalveolar lavage, skin biopsy, bone marrow biopsy which were all reported as unremarkable. He had a computed tomography (CT) scan of the chest which showed bilateral ground glass opacities predominantly in the upper lobes with increased nodularity and small-volume mediastinal lymphadenopathy (Figure 1). Laboratory tests were unremarkable except elevated serum lactate dehydrogenase (LDH) of 1491, C-reactive protein (CRP) of 39.8 and erythrocyte sedimentation rate (ESR) of 23. Eventually, video-assisted thoracoscopic surgery (VATS) resection was performed (wedge resection from right upper, middle, and lower lobes) and submitted for histopathological analysis.

## 2. Microscopic Description

**Figure 2:**
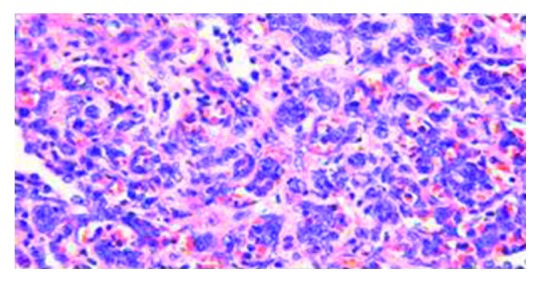
Atypical lymphocytes in the vessels (100x).

**Figure 3:**
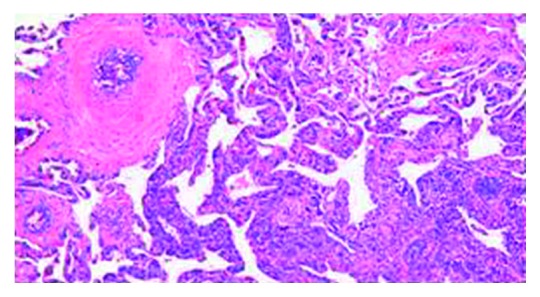
Hematoxylin-eosin staining atypical lymphocytes in the capillaries (20x).

**Figure 4:**
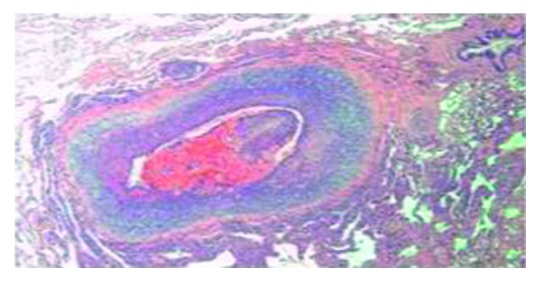
Hematoxylin-eosin staining showing angiotropism of medium size vessel.

**Figure 5:**
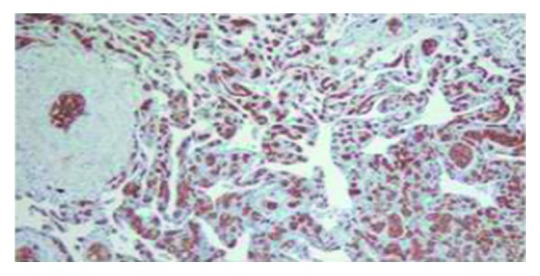
CD20 immunostaining showing intravascular atypical lymphoid cells.

**Figure 6:**
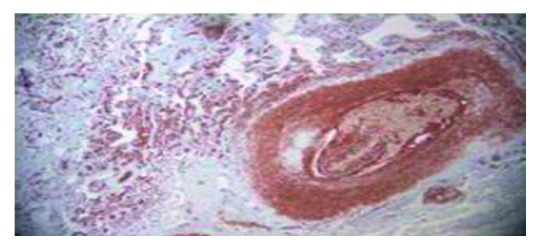
CD20 immunostain showing angiotropism of medium size vessels by atypical lymphoid cells.

Pathologic examination of the wedge resection of the lung showed expanded pulmonary interstitium by atypical, discohesive lymphoid cells within alveolar capillaries on routine hematoxylin and eosin staining (Figures 2 and 3). The distribution was patchy with areas of unremarkable lung parenchyma. The lymphoid cells showed irregular nuclear contours, vesicular chromatin, and prominent nucleoli. These atypical lymphoid cells also demonstrated angiotropism/angioinvasion of the medium sized pulmonary vessels (Figure 4). The walls of the pulmonary arteries showed heavy atypical lymphoid infiltrate without any fibrinoid necrosis/nuclear dust or RBC extravasation. A few capillary sized vessels showed tumor microthrombi. Immunohistochemical (IHC) staining (DAKO platform) showed that the atypical lymphoid cells were positive for CD20 (Figures 5 and 6), CD79A, Pax5, BCL2, MUM1, BCL6 (more than 30%), and CD10 (focal; less than 10%). Immunostain Ki-67 showed a proliferation index of 60%. IHC staining was negative for EBV-LMP, cyclin D1, CD30, CD23, melanoma cocktail, HMB45, and S100 stains. Immunostain CD31 and D2-40 highlighted the capillary structures within alveolar septae involved by these large lymphoid cells.

The patient was diagnosed with intravascular large B-cell lymphoma- (IVLBCL-) activated B-cell (ABC) phenotype (based on the immunohistochemistry staining pattern for CD10, BCL6, and MUM1 as per Hans Algorithm) [1].

## 3. Management

The patient underwent chemotherapy with CHOP-R (rituximab, cyclophosphamide, doxorubicin, vincristine, and prednisone) and remains in clinical remission twelve months after initial diagnosis.

**Figure 7:**
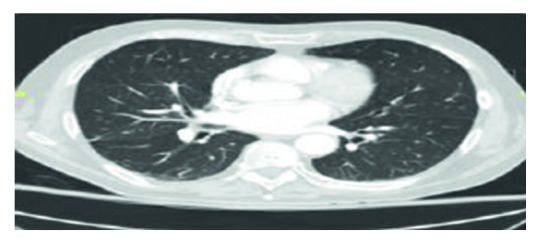
Chest CT scan 3 months posttreatment showing interval improvement of bilateral opacities.

A follow-up CT scan of the chest at three months posttreatment showed improvement of the ground glass opacities with no further axillary, hilar, or mediastinal lymphadenopathy (Figure 7) and a positron emission tomography-computed tomography (PET/CT) scanning performed 6 months after the diagnosis showed complete response with no residual disease.

## 4. Discussion

By the new World Health Organization (WHO) classification, IVLBCL is a rare type of extranodal large B-cell lymphoma characterized by the selective growth of lymphoma cells within the lumina of vessels, particularly capillaries, with exception of larger arteries and veins [2]. It is a rare and aggressive variant of lymphoma with little or no parenchymal involvement. The mechanism of selective intravascular location of IVLBCL is largely unknown. Several studies showed that the defective interactions between lymphoma cells and vessels may play a role in the pathogenesis of this disorder [3].

The clinical manifestations of IVLBCL are highly variable and mimics symptoms and signs of the organ affected. There are no pathognomonic clinical, laboratory, or radiological signs of IVLBCL. The disease most commonly affects the skin and central nervous system but may involve other organs, such as the lung, liver, kidney, adrenal gland, and prostate [4]. The most common clinical manifestations are neurological abnormalities resulting in progressive dementia and multiple neurologic deficits. Skin is the second most common site of involvement in the form of cutaneous plaques and nodules [5]. According to previous studies, CNS and skin involvement are commonly observed in Western countries, whereas hemophagocytic syndromes including fever, hepatosplenomegaly, and thrombocytopenia and bone marrow involvement have been described in Asian countries [6]. Although autopsy findings indicate that lung involvement in IVLBCL is relatively frequent (approximately 60%), predominant or primary presentation in the lung is rare. The diagnosis of pulmonary IVLBCL is frequently difficult to make because the clinical and radiographic findings are often nonspecific. The majority of cases with lung involvement show diffuse interstitial infiltrates, pleural effusion, signs of pulmonary hypertension, or consolidation [7].

Our case showed diffuse pulmonary ground glass opacities on CT scan with neurological symptoms and was finally proven to be IVLBCL on lung wedge resection.

The phenomenon of angiotropism/angioinvasion of the medium sized vessels has not been reported before in cases of pulmonary IVLBCL. Angiotropism is frequently seen in cases of central nervous system lymphomas. Rubinstein et al. in their paper said that the brain is the only tissue environment in which lymphomas cells accumulate around blood vessels [8].

IVLBCL must be kept distinct, by definition, from other lymphoproliferative disorders showing angiotropism, such as lymphomatoid granulomatosis and T-cell/natural killer-cell lymphomas of nasal type. These neoplasms typically involve, at a variance with IVLBCL, the vessel wall, whereas an intraluminal distribution of lymphoma cells is seldom observed. Although the term angiotropism has been ambiguously used in the literature also for describing cases of IVLBCL, this term should be disregarded whenever one refers to IVLBCL [9]. Occasionally, solid lymphomas may have an intravascular component. There are no randomized, controlled trials comparing treatments in IVLBCL. Most authors recommend an anthracycline-based chemotherapy regimens, such as cyclophosphamide, doxorubicin, vincristine, and prednisone with the addition of the monoclonal antibody rituximab [10]. The difficulties and delays in diagnosis often result in poor prognosis.

## 5. Conclusion

Intravascular large B-cell lymphoma is diagnostically challenging yet treatable disease. Correct pathology diagnosis in a fast and timely manner is crucial in patient management. These cases clinically mimic the signs and symptoms of the organ they affect with no obvious hematological symptoms, making them clinically challenging to diagnose. In our case, the patient had clinical and radiologic findings of interstitial lung disease. When the lung wedge biopsy was performed, an accurate diagnosis of IVLBCL was made.

Although a rare disease, our case should raise the possibility for consideration of IVLBCL in the differential diagnosis of interstitial lung diseases. Another major finding in this case is the angiotropism of the medium sized vessels, which has not been previously reported in IVLBCL of the lung.

Lung wedge resections along with CT scan chest findings with proper pathology workup led to the correct diagnosis in our patient, which enabled him to receive life-saving chemotherapy. Timely and appropriate biopsies avoid erroneous diagnosis and help and treat the patient accurately in a timely manner.
